# Optimizing the thermophysical qualities of innovative clay–rGO composite bricks for sustainable applications

**DOI:** 10.1038/s41598-023-48966-w

**Published:** 2023-12-07

**Authors:** Wafaa Soliman, M. Abdelhamid Shahat

**Affiliations:** 1 Geology Department, Faculty of Science, Sohag University, Sohâg, Egypt; 2 PV Unit, Solar and Space Research Department, National Research Institute of Astronomy and Geophysics (NRIAG), Helwan, Cairo, Egypt

**Keywords:** Climate sciences, Materials science

## Abstract

This work concerned the development of a unique reduced graphene oxide (rGO) nano-filler to provide innovative opportunities in enhancing the thermophysical performance of clay composite bricks. Whereas, a series of clay–rGO composite bricks were produced, doped with various levels of rGO nanosheets (i.e., 0, 1, 2, 4, and 6 wt% clay). Each clay–rGO composite’s microstructure, shrinkage, morphology, density, porosity, and thermophysical characteristics were carefully investigated, and the thermal conductivity performance was optimized. Incorporation of different levels of rGO NPs to the clay matrix allowed all the peaks intensity to rise relative to the untreated one in the XRD pattern. Meanwhile, the inclusion of these doping resulted in a grew in the crystallite sizes and apparent porosity within the compositions. In this vein, shrinkage fracture of fabricated brick composites varied depending on dopants type and levels during the drying and firing processes. Moreover, there are some changes in chemical compositions, as well as wave shifts, suggesting that functional groups of rGO may have contributed to partially introduce carbonyl groups in clay–rGO composites. Besides, the porous topography and bulk density improved rapidly with respect to the plane of the rGO nanosheets within the composites. The differ-dense microstructure displayed in the SEM micrographs supports these outcomes. Remarkably, clay–(4%)rGO compound not only has an optimum thermal conductivity value (0.43 W/mK), but it also has a high heat capacity (1.94 MJ/m^3^K). These results revealed the exceptional features of rGO sheets such as large surface area with high porosity within the modified clay composites.

## Introduction

Clay materials, which are available in a variety of mineralogy compositions with outstanding performance, present special physicochemical features^1^. Aside from being readily available and simple to use as well, it requires very little energy for its extraction, manage, fabricate, and transport. In addition to offering continual recirculation and interior comfort, these geotextiles are effective humidity and temperature controllers^2^. Therefore, these unique characteristics of clay materials allow them to be suited for numerous practical uses while protecting the environment, such as clay bricks^3,4^. Clay bricks have long been used as a building material and are regularly employed for creating walls, pavements, and other construction components^5^. Along with a wide range of other uses, including industrial, agricultural, and remediation of the environment^6^. Nevertheless, some studies have attempted to improve the effectiveness of building bricks based on a non-clay materials^7–9^. Whereas, paper bricks were produced as a replacement construction material by Akinwande and et al.^7^, using waste paper pulp, fine sand, regular Portland cement and banana fiber blended in different amounts. The thermal conductivity rose depending on fiber dosage (0, 0.5, 1.0, 1.5, 2.0, and 2.5 wt%). Zuraida et al.^8^ looked at recycling used diapers as an alternative resource for the structural and architectural parts of a buildings, while Ashish Soni et al.^9^ suggested combining waste plastics with silica sand to create thermoplastic hybrid tiles for flooring. In a similar vein geopolymers are utilized in low-CO_2_ bricks, cement, concrete, and 3D mesh for fire and heat-resistant fibres, contingent upon the structural chemical network. Utilizing the silicon phosphate modification, Qinglei Sun and et al.^10^, formed a novel waterproof geopolymer that bonds to alumina and quartz glass substrate. The microhardness and adhesion strength of the resultant geopolymer adhesive were optimized with regard to the quantity of Si_3_(PO_4_)_4_ loading (i.e., the mass ratio of metakaolin, water glass, and silicon phosphate of 0.48: 1: 0.08). Meanwhile, Qinglei Sun and et al.^11^ developed a geopolymer composites composed of metakaolin and surface-modified hexagonal boron nitride that can generate 3D forms in order to ease direct inking. Madiha Ahmad and Khuram Rashid^12^ produced a novel clay-based geopolymer brick using a variable ratio of fly ash to clay content (25–75%) along with two molding pressures (20 and 40 MPa). The physicomechanical, geopolymerization rate, and microstructure features of the developed clay compounds were optimized, as were the compressive strength and density, porosity, and water absorption of carried out bricks.

Clay and sand mixtures are traditionally formed, dried, and fired at temperatures between 900 and 1100 °C to produce bricks^13^. Whereas, the vitrification of minerals at high temperatures results in the formation of glass phases that densify the brick structure. Sequentially, the physicochemical changes that occur during the firing process have an impact on the thermal and diffusivity/conductivity features of a brick’s performance^1^. Among these physicochemical changes are water removal, dehydroxylation, crystalline transformations (i.e., kaolinite to mullite minerals), glassy phase growth, and mineralogical modifications. These variations may possess a direct influence on the porosity and density nature of the material, thereby affecting the thermal and diffusivity/conductivity qualities of the final brick’s performance. A lot of research has been done on the thermal conductivity of clay–based substances, especially as these components are thermally modified and assembled for practical application^5,14^. Thermal conductivity is greatly impacted by the ultimate porosity, bulk density, raw material type, and temperature at which solid phase transitions arise. The way in which each of these parameters affects conductivity is readily apparent by the vibration of atoms and molecules inside their lattices^15^. Bricks of clay–based compound frequently exhibit values below 1W/mK^16^. Meanwhile, the internal energy of clay bricks and the continuous movement of particles with quick vibrations both serve as indicators of specific heat capacity, which is correlated to thermal conductivity^15,17^. Nevertheless, to improve particular properties of clay bricks, different dopants are added to the clay mixture^18–22^. Especially after the brick firing process, it results in more durable and strong materials. Further, the raw clay’ compositions, the additives existence, and the level of chemical oxidation or reduction all affect the clay mineralogical changes during burning^23^. Wang et al.^18^ improved the thermal diffusivity of burnt brick by incorporating vermiculite (a hydrous clay mineral) within the clay composition, whilst Lawanwadeekul et al.^19^ improved the porosity and strength of bricks by altering the corn cob/waste glass levels. Parallel to this, Gencel and et al.^20^ constructed burnt clay bricks containing varying amounts of industrial slags to enhance porosity and thermal conductivity characteristics (0.93–1.10 W/mK).

In line with the same objective and to fill specific research gaps, the physiochemical properties of clay bricks can be changed by adding different types of nanofillers to the clay matrix. These parameters include surface area, porosity, density, and others^24^. Among these substances are rGO NPs, possessing distinct microstructure features and large surface area, resulting in outstanding thermophysical properties^25^. This is mainly caused by a large amount of hydroxyl groups and other functional groups bonded to oxygen atoms on the surfaces of these nanosheets^5^. The hydrophilic and hydrophobic sites within the clay structure can be rearrangement by these additions, which allow to control the form and direction of water flow^6^. Therefore, once added to water, high-quality bricks can be created. In a similar vein, the clay–rGO composite bricks produced by incorporating rGO sheets into the clay matrix reveal good thermal efficiency and considerable conductivity. However, increasing the amount of the two-dimensional (2D) rGO results in closer sheet bonding as well as excellent dispersion over the whole composite^5,26^. These improvements might be related to rGO carbonyl functional groups, chemical association, cation exchange and establish hydrogen bonding on the metal oxide surfaces with the clay compositions^27–29^. Additionally, the formation of these groups is markedly affected during the firing process at 1100 °C, as physicochemical activities including the disintegration of quartz proceed once the burning increases to 800 °C before vanishing at 1100 °C, supporting the evolution of the mullite element^30^. The nanosheets subsequently turn active and uniformly distributed throughout the firing process, penetrating the clay layers isotopically with the help of free hydroxyl bonds. These physicochemical activities provide these compounds with unique qualities including a large number of functional groups, a wide expanse of surface, and substantial charged-carriers movement. Therefore, burnt brick compounds with remarkable thermophysical qualities are produced^31,32^. In particular, in the planar structures of rGO sheets, a high temperature stimulated an active-sites in both the top and bottom surfaces of the rGO sheets, bringing them into close contact with the host substance^33^. Additional potential locations for beneficial physicochemical changes are provided by the enlarged surface areas, which in turn enhance the bonding between rGO and host materials. Meanwhile, variations in functional group levels on rGO sheets greatly influence typical van der Waals bonds, controlling water distribution^34^. In addition to the aforementioned physiochemical features, this nanofiller is beneficial economically since it is composed of typically available, cost-effective natural minerals along with is readily generated using straightforward techniques and equipment^8^. The basic part of fired clay bricks (clay) is simple to obtain since there are plenty of clay resources accessible worldwide, reducing the requirement for transportation^19^. In this regard, there are millions of tons of abundant clay deposits widely distributed throughout Egypt. In particular, clay deposits amounting to millions of tons are widespread throughout Egypt^35^. Meanwhile, rGO fillers are frequently economical in both mining and production procedures since they are extracted from abundant natural resources and mines, like graphite, a form of carbon. Regarding the energy losses in conventional clay bricks employed in diverse purposes (e.g., buildings), the thermal performance of these unique hybrids is optimized whilst keeping the economic feasibility of fired clay bricks.

Although the above-mentioned features of rGO additives, there are insufficient studies on their influence on the thermal behavior within clay composites. According to our knowledge, innovative clay–rGO nanocomposites were designed for the first time in the current study to improve the thermophysical performance of clay bricks. These composites contained various levels of rGO nanosheets (i.e., 0, 1, 2, 4, and 6 wt% clay). rGO nanosheets were developed particularly to provide a wide range of functional groups (e.g., C=O, carbonyl and SP^2^ bonds) which create unique bonds with the pristine clay elements, enabling regulation of the water level inside the combined product and therefore boosting thermal activity. At this stage, toxicity maintains below the permitted range, and the bricks are highly responsive to their production activities. Consequently, the utilization of these smart substances opens up prospects for improving thermophysical features in future clay-based brick usage.

These innovative brick composites will also find future use in water-cooled kiln walls, generators, mufflers, and coke oven walls—all places where efficient thermal control is essential^36^.

## Experimental

### Materials and analytical methods

Precursors for the synthesis of rGO nanosheets included sulfuric acid (H_2_SO_4_), phosphoric acid (H_3_PO_4_), graphite powder, potassium permanganate (KMnO_4_, 99%), hydrochloric acid (HCL; 37%) and hydrogen peroxide (H_2_O_2_; 30%) were used as intermediates. On the other side, clay minerals were gathered in fine grains from the Kharga Oasis, where the clay is formed up of different crystalline mineral compositions. The Kharga Oasis, situated in the Egyptian Western Desert (Lat. 25.46–25.78  N and Long. 30.54–30.91  E), is one of the main depressions. The oasis is bounded on the north and east by a limestone ridge, the precipitous rocks of which form a sharp barrier to the valley floor^37^. However, as you move south and west, the depression’s bottom slowly blends with the Nubian Sandstone desert^38^. The Nubian Sandstone series primarily makes up the Kharga Oasis’s base and subsurface succession^39^.

### Powder synthesis of rGO NPs

A modified hummers method was used to create highly qualified graphene oxide (GO) nanosheets. In addition, the level of functional groups was reduced using an in-situ oxygen plasma surface modification technique to create rGO nanosheets, as described in details in our previous work^40^.

### Synthesize of clay–rGO nanocomposite bricks

**Figure 1:**
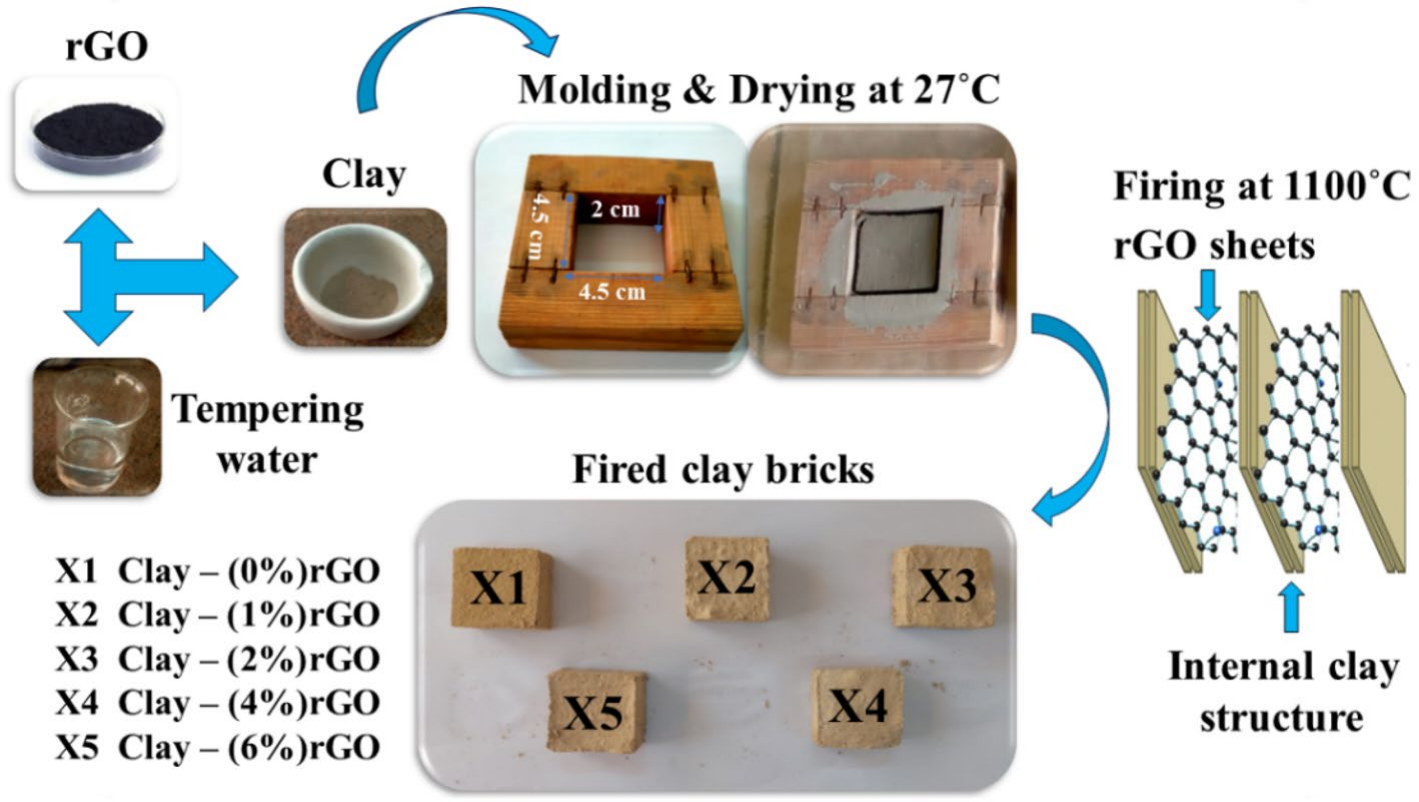
Clay-rGO composite bricks during the molding and firing process at at 1100 °C.

A series of clay–rGO composite bricks were produced, doped with various levels of rGO nanosheets (i.e., 0, 1, 2, 4, and 6 wt% clay). Figure 1 displays a sequence of clay–rGO composites molding and firing processes, as well as a rGO sheets dispersion diagram within the clay internal structure. Whereas, each hybrid block is composed of up of 50 g of solid components mixed with 40 ml of tempering water. The clay compound’s test portions were hand-shaped in a hardwood frame laboratory molding (4.5 cm × 4.5 cm × 2cm). These engineered hybrid materials performed well during molding and dried safely with no obvious cracks. To ensure there was no moisture present, the newly constructed raw bricks were naturally dried at RT for three days before being fired for 4 h at 1100 °C. Due to the abundance of alkali oxides inside the burned samples in this situation, burning at 1100 °C assures higher vitrification, giving the bricks more resistant to water penetration and therefore fewer prone to humidity associated weathering deterioration^20^.

### Ethical approval

This article does not contain any studies with human participants or animals performed by any of the authors.

## Characterization of the composite bricks

X-ray Diffractometry (XRD) with a Cu target (Model D8 is an ADVANCE, Bruker) was used to evaluate the mineralogical composition characteristics of the unmodified and modified clay composites. Patterns were seen between 4 and 70° 2θ. Further, Fourier transform infrared (FTIR) spectroscopy (Jasco Model 4100_Japan) was utilized to examine the structural changes caused by chemical modifications of clay substances. At RT, the FTIR measurements yields observations with a resolution of 4 cm^−1^ in an area of 4000–400 cm^−1^. Meanwhile, using a high-resolution scanning electron microscopy (SEM, JEOL JEM-2100) (Japan), the surface morphology of each of the clay–rGO composites was evaluated. Drying and firing shrinkage behaviour were evaluated by estimating the variation in dimensions of the clay brick after drying and firing, respectively, at room temperature. A SETARAM LABSYS Evo thermogravimetric analyzer (TGA) 1600 was employed for the thermal stability investigation, which was conducted in the 23–1000 °C temperature range. In the air surroundings, both cooling and heating rates were 10 °C/min with an isothermal accuracy of $\pm$ 1 °C. Thermal characteristics were also recorded with a hot disc thermal constants equipment (heated Disc TPS 2500 S, Göteborg, Sweden). Whereas, this Transient plane source (TPS) method is the most accurate for investigating thermal transport characteristics. Moreover, physical observations of bulk density as well as apparent porosity were performed via the Archimedes technique.

## Results and discussion

### XRD analysis

**Figure 2:**
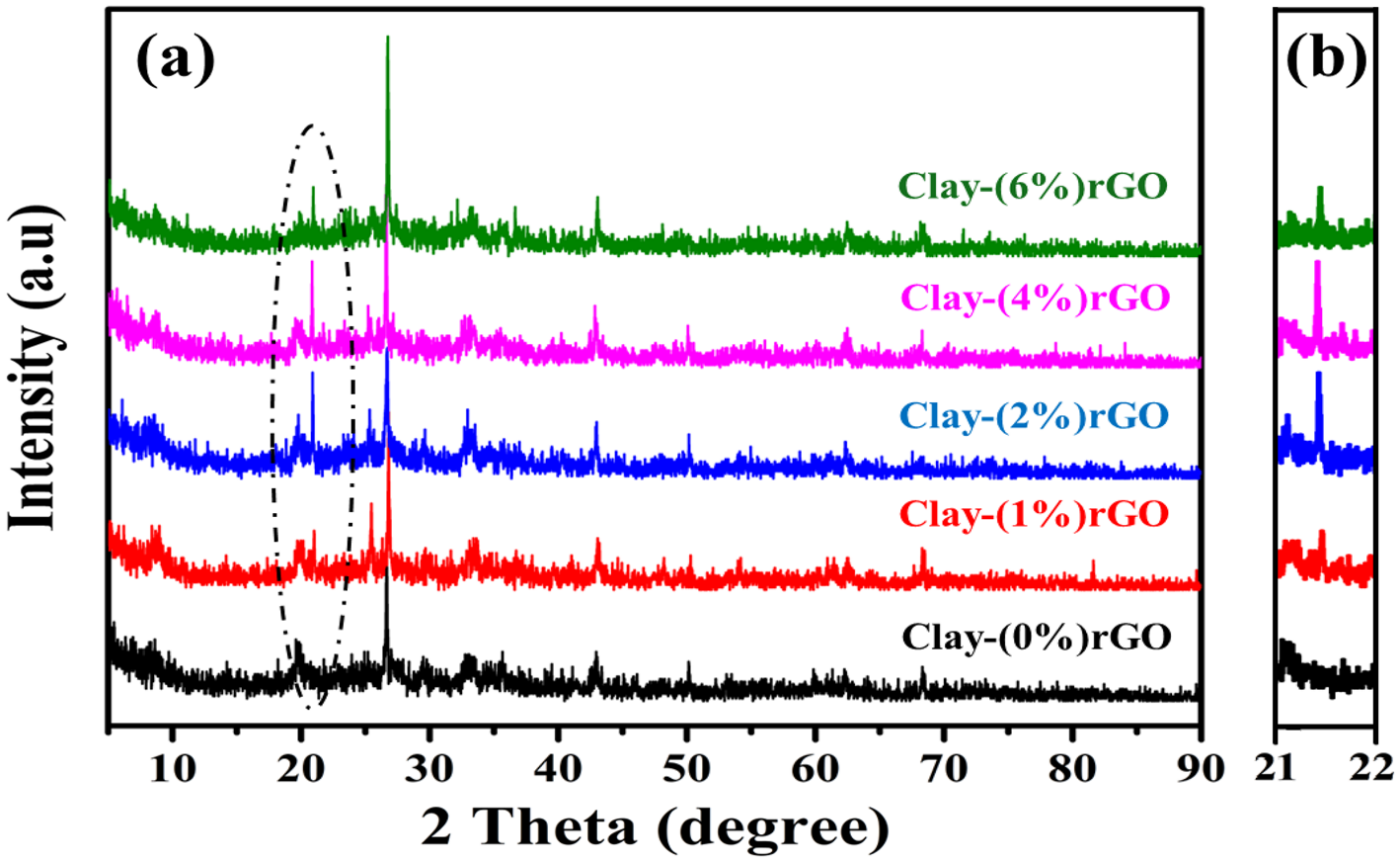
X-ray diffraction profiles of clay–rGO composites prepared at different concentrations of rGO NPs (0, 1, 2, 4, 6 wt% clay).

The XRD technique was employed to investigate the structural changes in the clay structure caused by impregnation with several concentrations of rGO nanosheets (i.e., 0, 1, 2, 4, and 6 wt% clay). Figure 2a,b depict XRD profile peaks of as-synthesized clay–rGO composites in the (4°–80°) and (21°–22°) ranges, respectively. The pristine clay composition contains a variety of mineral types and amounts, which are summarized in Table 1. Consequently, unmodified compound exhibits distinct reflections at 2θ values of 29.4°, 43.3°and 48.5°, which correspond well to calcite (COD card No: 1010928). Illite (COD card No. 9013732) and quartz (COD card No. 1011176) both had diffraction peaks at (2θ = 50.3°, 60.2°), and (2θ = 26.5°), respectively. Additionally, kaolinite (COD card No. 9009230) was found at 2θ of the following locations: 12.2°, 20.2°, 24.8°, and 39.4°, as well as the phyllosilicate mineral (2θ = 12.5°)^41^. Meanwhile, 2θ = 21.7°, 23.6°, and 27.7° were detected for feldspar at^41,42^. Thereafter, incorporating different levels of rGO NPs to the clay matrix allowed all the peaks intensity to rise relative to the untreated one. Meanwhile, the inclusion of these doping resulted in a newly induced peak at 21.5° (JCPDS card No: 752078), and its intensity grew as more rGO load within the clay–rGO lattice structure. This varying behaviour is clearly depicted in Fig. 2b, which use a zoomed image of peak at 21.5°, respectively. This trend confirms that rGO was effectively combined into the clay support, as well as causing structural defects and disorders in the clay’s crystal structure^43^. Similar findings from previous studies were reported^43,44^.

**Table 1:** Mineralogy of the raw material.

Bulk mineralogy	Phyllosilicates (45%)	Quartz (8%)		Feldspars (18%)		Calcite (9%)	Dolomite (20%)
Clay fraction (˂ 2 µ)	Smectite/illite mixed layers (S/I) (70.8%)				Kaolinite (29.2%)		
Grain size	Sand (2%)		Silt (78.8%)			Clay (19.2%)	

However, the level of rGO sheets employed had an effect on the calculated crystallite size of clay–rGO compounds. Where, using Scherrer’s formula, the combination’s crystallite size was estimated^45^:

1 $$ D = \frac{0.94 \lambda }{{\beta \cos \left( \theta \right)}} $$

where λ is the wavelength, β is the full width at half maximum of the peak, and θ is the diffraction angle. The crystallite size of the pristine substance was 23.7 nm and gradually grew to 24.3, 27.5, and 35.4 nm with respect to the rGO level up to 1%, 2%, and 4%, respectively, before reducing to 34.2 nm at 6%-rGO. This can be clarified via the reactions that generated after the dopant was introduced, which supports the substantial physiochemical variations of the clay on the rGO surfaces.

### FTIR spectra

**Figure 3:**
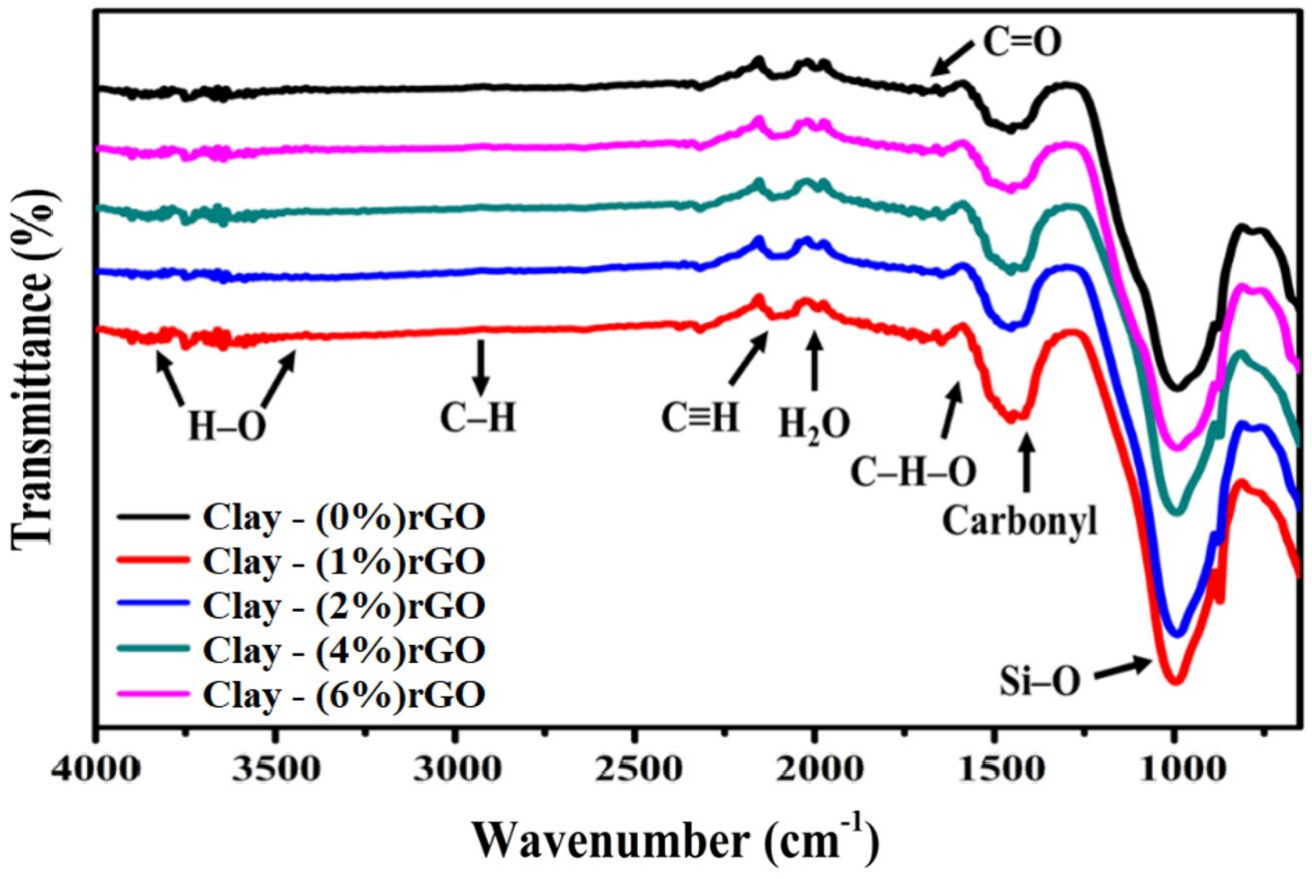
FTIR spectra of clay–rGO composites prepared at different concentrations of rGO NPs (0, 1, 2, 4, 6 wt% clay).

FTIR spectra were used to evaluate the effect of rGO concentration on the chemical compositions of clay–rGO composites, as seen in Fig. 3. A wave at 3854–3354 cm^−1^ associated with the H–O expansion vibration of H-bonded adsorbed water molecules in pure clay was detected. Then it progressively vanished in the composites due to the non-polar property of rGO, which reduced the water absorption^27,46,47^. The short band at 2922 cm^−1^ is associated with C–H stretching mode and indicates that some organic contribution is present^48^. In addition, the signals at 2184 cm^−1^ and 2020 cm^−1^ are assigned to C≡H stretching and H_2_O, respectively. Meanwhile, the small signals recorded at 1725 and 1625 cm^−1^ may be attributed to the C=O and C–H–O, respectively^49^. Besides, carbonate species, Si–O out-of-plane stretching, and amorphous meta kaolinite structural bonds were situated at 1416, 991, and 872 cm^−1^, respectively^50,51^. Similar findings have been reported in previous studies^47,52^. Moreover, there are some changes in peak intensities and wave shifts with respect to the plane of the rGO nanosheets within the composites. Whereas, modified samples revealed a slight shift in absorption peaks (3354, 2922, 141, 991, 872, 797, 776 cm^−1^) towards higher wavenumber values as compared to the unmodified sample. The peaks at 797 and 776 cm^−1^ were attributed to the symmetrical and asymmetrical stretching vibrations of the quartz Si–O–Si bonds between the tetrahedron^27^. The shift in the tetrahedral arrangement, combined by a decrease in the degrees of crystallinity in the tetrahedral plate, may be responsible for the deviation of the Si–O–Si bonds and the transition to higher frequencies^53^. However, the peaks at 2184 cm^−1^ and 2020 cm^−1^, had a movement towards low wavenumbers, indicating the establishment of hydrogen-bonding interaction between clay and rGO NPs^54^. Along the same lines, the observed peak intensity of the composite vibration increased relative to the untreated sample and increased progressively as the compound’s rGO level rose. Wherein, the strength of the carbonyl peak at 1416 cm^−1^ rose as the amount of rGO increased, suggesting that functional groups of rGO may have contributed to partially introduce carbonyl groups in clay–rGO composites^49^. These experiments revealed that rGO and clay may be combined successfully.

### Morphology

**Figure 4:**
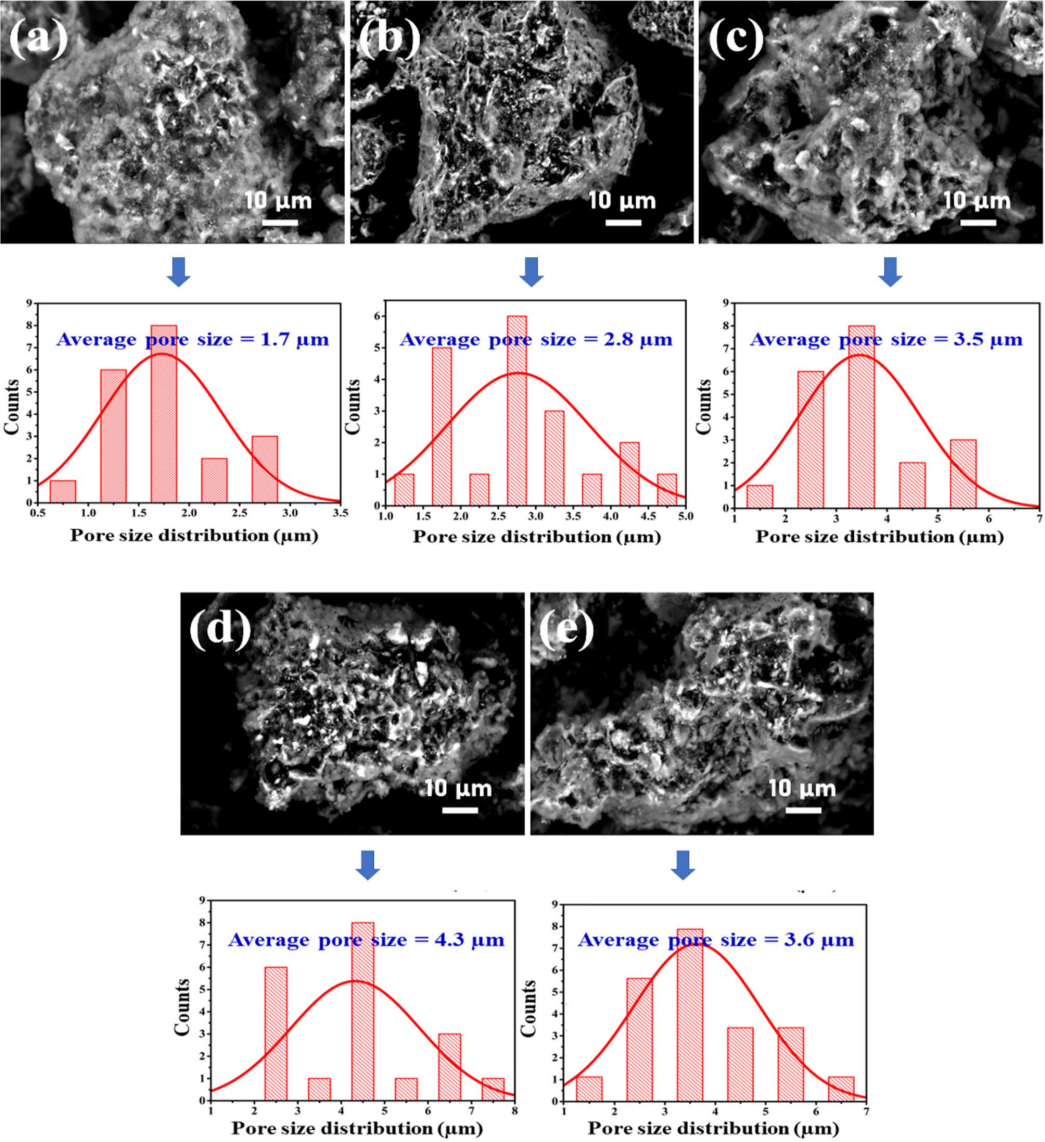
(**a** – **e**) SEM micrographs and statistical histograms of pore size distribution of untreated and treated nanocomposite clay using various ratios of rGO NPs.

Figure 4 depicts SEM micrographs and pore size distributions of pristine and treated clay–rGO composites with various rGO additions. Depending on the doping level, the porous topography of the clay–rGO composites can be observed. Whereas, Fig. 4a presents the surface features of the pristine fine-pored clay. Besides, comparable structures are detected in Fig. 4b–e in clay and rGO nanosheets which were incorporated differently. Meanwhile, several wrinkled and crumpled structures were observed in the clay composites. This property of clays is crucial in preventing undesired restacking and aggregation of individual nanosheets^49^. Furthermore, the findings indicate that the rGO functional groups and the clay matrix adhered well to each other, resulting in hydrogen bonds formation and uniform distribution of rGO inside the clay structure^55^. The structure of the composites also expanded at higher rGO levels, revealing an amorphous structure with more micropores, multiple bonds, and increased internal energy^56^. In addition, the average pore sizes for the pure and modified clay–rGO composites were statistically estimated. Whereas the average pore size estimated for pure clay was 1.7 µm and was enhanced to 2.8 µm by incorporating 1%-rGO nanosheets within the clay matrix. Thereafter, pore size values improved systematically as rGO content increased, achieving an optimum value of 4.3 m in clay–(4%)rGO composites. Moreover, increasing the quantity of nanosheets in the composites resulted in the opposite behaviour, with the estimated value decreasing to 3.6 µm in the clay–(6%)rGO composite. These observations might be explained by the creation of new intermolecular covalent bonds and hydrogen bonds between clay–H_2_O and rGO functional groups^14^. These bonds influence the rate of hydration crystal growth and lead to the uniform dispersion of nanosheets inside clay–rGO composites^57^.The result was a notable improvement in the surface roughness and thermal characteristics of these composites as a result of the miscibility of nanosheets.

**Figure 5:**
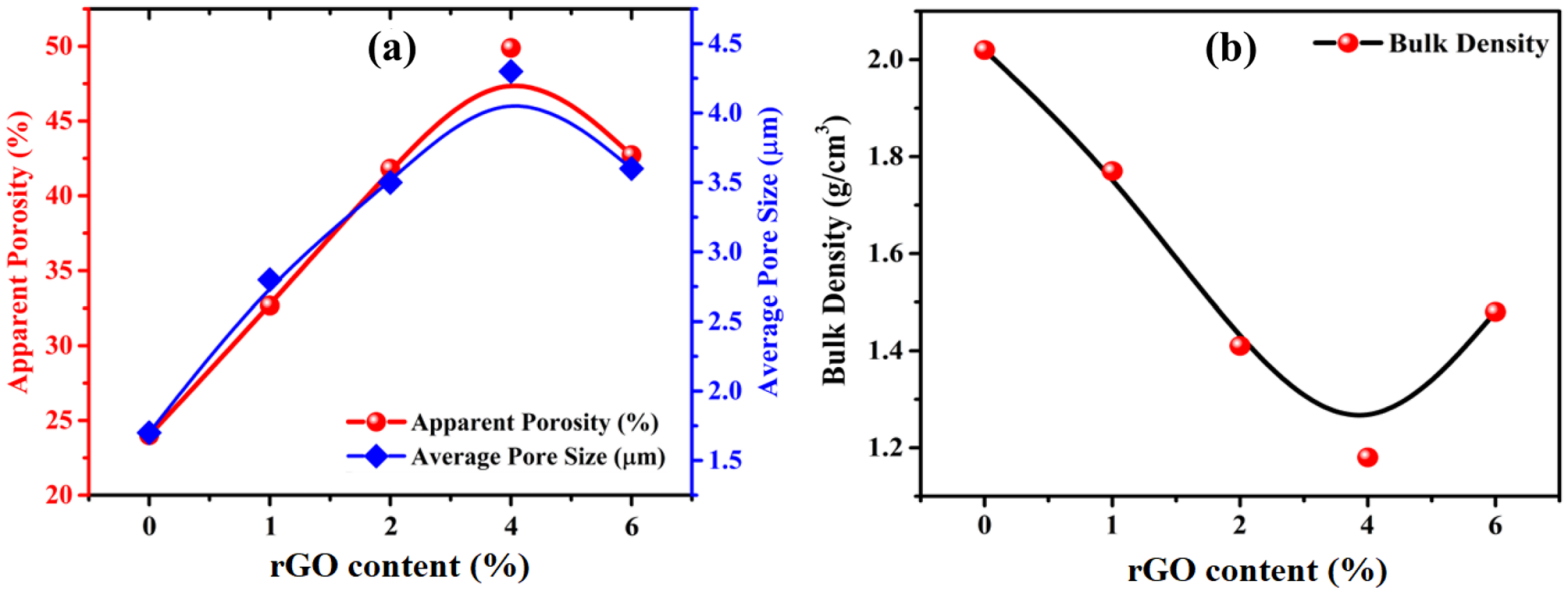
(**a**) Porosity, (**b**) Bulk density of clay–rGO bricks at different levels of rGO nanosheets.

In light of the importance of porosity, which is in alignment with this, the Archimedes technique was employed for calculating the apparent porosity and density characteristics of the created compounds (See Fig. 5). Since, they are one of the most important factors influencing thermal conductivity^2,58^. Pristine clay exhibited the lowest apparent porosity value (24.01%), that subsequently increased to 32.67, 41.81, and 49.89% with regard to the rGO load levels (1, 2, and 4%), respectively. Furthermore, adding more dopant nanosheets resulted in a reversal of the trend, with apparent porosity falling to 42.72% at the 6%-rGO sample. The variation in this pattern is closely correlated with the average pore size values derived from SEM micrographs and depicted in Fig. 5a. 4%-rGO nanosheets are clearly the most effective level to optimize the apparent porosity necessary for a good thermal conductor. Since during firing at 1100 °C, bricks with additives have a more porosity nature than ordinary bricks. This is due to the degradation of CaCO_3_ which leads to combustion of additives or pores. Meanwhile, as seen in Fig. 5b, the bulk density of the pristine substance was 2.02 g/cm^3^, then reduced to 1.18 g/cm^3^ with regard to the rGO level up to 4% before growing to 1.38 g/cm^3^ at 6%-rGO. The differ-dense microstructure displayed in the SEM images supports these outcomes. Moreover, the variations in the density trend are driven by the growth of finer particles, which in turn promote densification by raising the rate of atomic diffusion, leading to denser microstructures^59^. Meanwhile, the density of bricks containing additives reduces as a consequence of the chemicals’ propensity to decay during the combustion cycle^23,60^. An increase in porosity and a decrease in bulk density can have several of beneficial consequences on the performance as well as the features of burnt clay bricks. The benefits are as follows: lightweight bricks, moisture resistance, reduced shrinkage and cracking, lower thermal expansion, cost savings, energy-efficient burning, sustainability, sound and thermal insulation in buildings. Whereas, adding additional air pockets within the brick regulates temperature and reduces energy consumption, hence improving structural insulation. Before that, studies using various dopants, such as fly ash and TiO_2_ NPs, revealed a similar pattern of bulk density behavior^29,61–63^.

### Shrinkage behaviour

**Figure 6:**
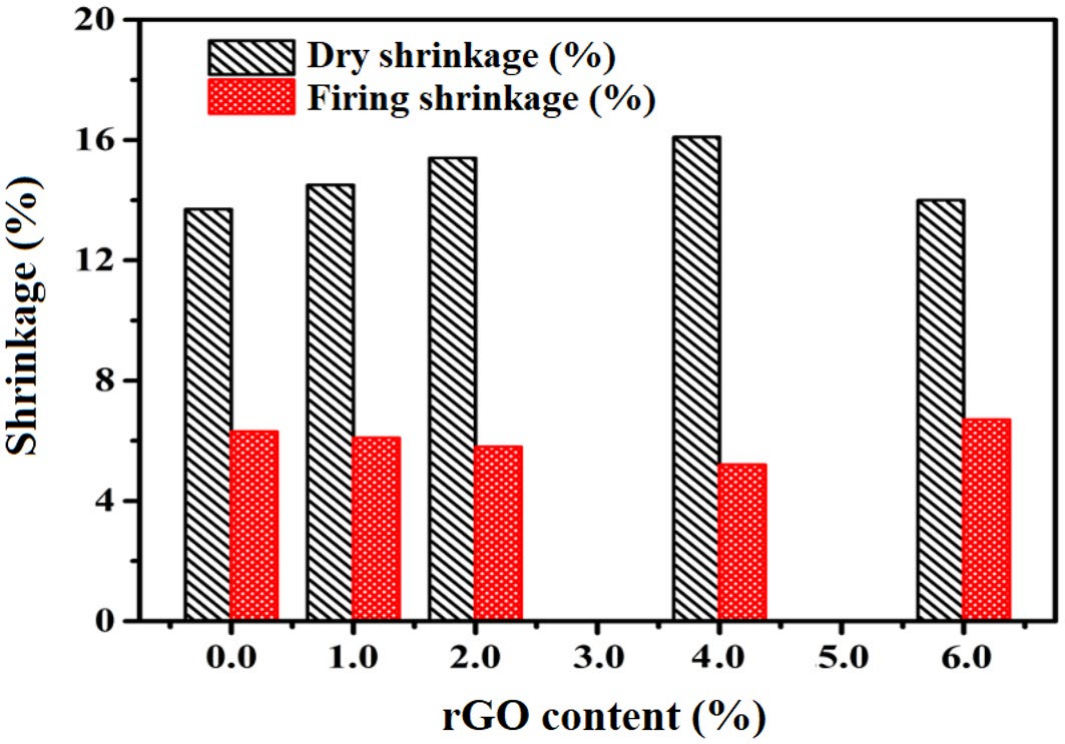
Drying and firing shrinkage of clay–rGO bricks at different levels of rGO nanosheets.

As demonstrated in Fig. 6, the shrinkage behaviour of the produced brick composites with regard to rGO levels varied during the drying and firing stages. In the first process, the orientation of the chips in soft mud bricks, that are formed by throwing clay into molds, is more random throughout the drying stage, and as a result, their behaviour is more isotropic. Once the formed brick dries, the volume reduces (by at least 6%) and some initial strength develops. As a result, the interior of the brick shrinks slower than the outside. Moreover, shrinkage cracks might appear if water evaporates too rapidly, reducing strength significantly^64,65^. Our literature states that high-quality bricks should shrink 10% when dried. Whereas, the samples exhibit acceptable drying shrinkage with undetected shrinkage fractures. Herein, the dry shrinkage systematically increased from 13.7% of the pristine compound to 16.1% with the addition of 4%-rGO, respectively. The existence of carbonyl functional groups and hydroxyl groups in rGO may be responsible for this outcome because they interacted with clay structure through ionic exchange, hydrogen bonding, and chemical association^28^.

On the other hand, the calcination process, which involves heating composite bricks to a high temperature, causes some minerals in the clay matrix to melt, changing the volume and strength of the brick. Further, as the volumetric mass increases, the brick becomes stronger while maintaining its weight. According to the fact that quality bricks have a firing shrinkage of less than 8%, all of the molds in this study had good firing shrinkage.

*At the firing stage in this work,* the shrinkage attained was 6.3% of the pristine combination and gradually decreased to an optimal value of 5.2% with the addition of 4%-rGO nanosheets. These enhancement might be ascribed to the hydrophobicity of graphene platelets and the nanofiller-matrix adhesion/interlocking caused by their wrinkled surface, which have better two-dimensional geometry^33,66,67^. Besides, these findings are in line with previous studies that suggested 1 wt% of rGO might enhance the toughness of clay–rGO composites. As, the sheets are connected, some of them overlap, forming strong plates that are firmly attached to the matrix^68^. Additionally, the removal of certain hydroxyl, functional groups, chemically mixed water, and the reduction of substances into ashes, in addition to other activities that clearly lower its volume, may also be directly contributing to the low values of firing shrinkage. Finally, these chemical changes and the repositioning of particles in the crystal lattice that occur during firing result in a more compact solid structure^65,69,70^.

### Thermogravimetric Analysis (TGA)

**Figure 7:**
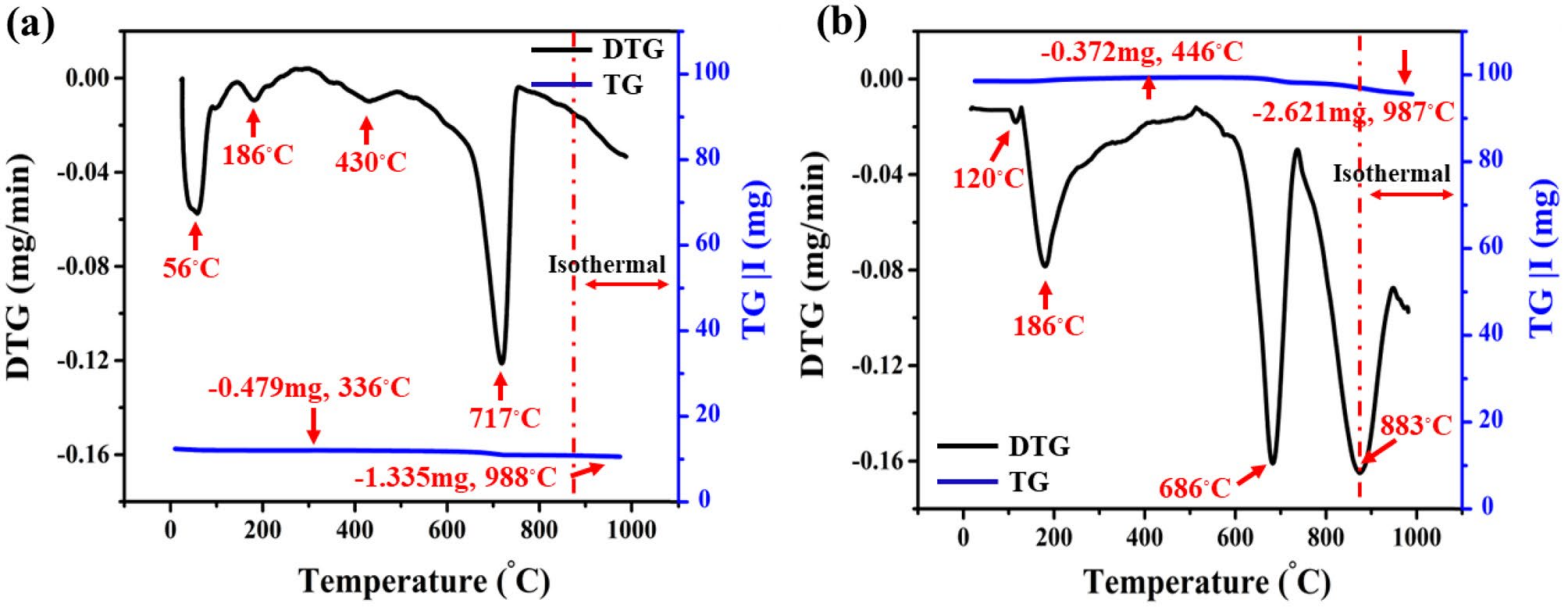
TGA and DTG curves for (**a**) unmodified and (**b**) rGO-treated clay composites.

TGA and DTG techniques were performed to investigate the thermal stability of the untreated and treated clay composites depending on the functionalization degree of rGO sheets, as seen in Fig. 7. The dehydration of clay minerals was caused by water molecules that were absorbed into the clay combinations, as seen in Fig. 7a,b. This included both physically received water and bound water associated with interlayer cations in the 25–300 °C range^71^. This resulted in a 0.431 mg and 0.312 mg drop in physical weight for the pristine and modified clay materials, respectively. The shift occurred due to the existence of rGO inside clay layers, thus leading to the inclusion of extra functional groups (including carbonyls (C=O) and carbon atoms SP^2^). In-situ XRD patterns provide practical support for this findings^72^. Besides, the pure substance recorded a small intensity of the DTG peak at 186 °C, however the clay–rGO instance exhibits a wide and high intensity of the peak. The evolution can be attributed to the presence of oxygen-containing compounds produced by rGO sheets, including carboxylates and epoxy, whose pyrolyzed releasing CO, CO_2_, as well as H_2_O vapour^73,74^. Likewise, the dehydroxylation of kaolinite is the main reason for the weight losses for both treated and untreated composites between 300 and 750 °C^75^. In turn, clay–rGO dropped 0.372 mg while pure clay lost 0.489 mg. It is plausible that additional clay minerals, such smectite and illite/mica, also undergo dehydroxylation within this temperature range and very marginally influence the weight reduction. A peak observed at 717 °C and 686 °C on both DTG curves is ascribed to the decomposition of calcite and the dehydroxylation of illite^76^. As for the clay–rGO composite, its DTG curve displayed an apparent peak at 883 °C with 2.621 mg weight loss, suggesting the most stable groups of nanofiller sheets—like carbonyl and quinone—were breaking down. The 10 °C/min ramp rate utilized in this investigation yielded results comparable to mullite or γ-alumina nucleation acceleration.

The residual weights of the pristine and modified clay substances during the 25–1000°C temperature range were 96.77% and 93.91%, respectively. This suggests that clay bricks containing rGO have a slightly greater weight loss. Therefore, the high thermal stability of these fired clay bricks leads to great construction durability, thermal insulation, thermal shock resistance, and energy efficiency.

### Thermal Properties

The thermal features of clay bricks were significantly influenced by the level of rGO nanosheets incorporated. Table 2 addresses these modifications to the thermophysical performance, including thermal conductivity, thermal diffusion, thermal effusivity, and specific heat capacity. In particular, the thermal conductivity of the pristine clay was 0.32 W/mK, then gradually grew to 0.33, 0.35, and 0.43 W/mK as the rGO level rose to 1, 2, and 4%, respectively, before dropping to 1.38 g/cm^3^ at 6%-rGO. These thermal characteristic variations are prompted by the significant interaction between clay–H_2_O products and oxygen functional groups (epoxides, carboxyl’s, and hydroxyls) of rGO nanosheets^7^^,^^14,77^. Therefore, these interactions influence the rate of hydration crystal growth and result in a uniform dispersion of nanosheets inside the clay–rGO compound^57^. Moreover, to enhance heat conduction, significant thermal diffusivity values are also necessary. Therefore, a material with a high thermal diffusivity has physical relevance as a result of the rapid rate at which its temperature changes when heated^78^. Several previous studies revealed a similar pattern as it came to enhancing the thermal performance of fired clay bricks^7,18,20,21^. The inclusion of a variety of rGO nanosheets within a clay matrix raised both thermal diffusivity and thermal effusivity behaviour. Whereas, clay–(4%)rGO compound not only has an optimum thermal diffusivity value (0.46 mm^2^/S), but it also has a high thermal effusivity (863.88 Ws^1^´^2^/m^2^K). The relatively high thermal diffusivity of these samples enables heat to spread further and to be absorbed by the substance less^79,80^.

**Table 2:** Thermophysical characteristics of nanocomposite clay bricks with various levels of rGO dopant.

rGO content (wt%)	0	1	2	4	6
Thermal conductivity (W/mK)	0.32	0.33	0.35	0.43	0.38
Thermal diffusivity (mm^2^/S)	0.28	0.29	0.34	0.46	0.25
Thermal effusivity (Ws^1^´^2^/m^2^K)	602.87	756.17	812.88	863.88	790.26
Specific heat capacity (MJ/Kg K)	1.15	1.31	1.62	1.94	1.5

Additionally, it is remarkable to note that the heat capacity of the clay–rGO composites rose (1.15–1.94 MJ/m^3^K) as the GO amount (0–4%) grew, then reduced to 1.5 MJ/m^3^K with the addition of 6%-rGO. The main explanation for these findings is the existence of an appropriate level 4%-rGO sheets, which revealed exceptional features like large surface area with high porosity inside the modified clay composites. Whereas, at lower levels of rGO (1–4%), ion exchange arises with alkaline clay pore solutions of charged ions such as Ca^2+^, Mg^2+^, Na^+^, and OH^−^, and the distinctive surface area of the rGO sheets efficiently permitting liquid absorption of the interlayers^57^. Nevertheless, raising the level of 6%-rGO in clay formation may not promote their efficacy since excess sheets lead to enhance rGO agglomeration^81^. Aggregation enabled rGO to lose its intrinsic benefit of having a large specific surface area, thus it is unable to act as a site of nucleation which accelerates the hydration and porosity distribution of clay composites, and affects the thermophysical performance of clay matrix^82^. As a consequence, rGO agglomeration with small levels and an increased specific surface area is selected since it maintains its efficacy inside the structure and improves the performance of the clay compound. According to^83^, all of the specimens meet the required specifications since their thermal conductivity is below 0.6 W/mK.

## Conclusion

In this context, the internal clay structure was modified by impregnation with several concentrations of rGO nanosheets (i.e., 0, 1, 2, 4, and 6 wt% clay). The inclusion of rGO in clay matrix at varying proportion significantly affected the growth rate of hydration crystals that turns on the thermophysical properties of composites. Whereas, the compositions’ crystallite sizes, bulk densities, and porosity characteristics all improved as a result of the doping’s addition. Additionally, during the drying and fire activities, the behavior of shrinkage of produced brick composites changed according to the kind and concentration of dopants. therefore, these dopants level within the composite’s surfaces revealed their excellent compatibility and strong interfacial adhesion between clay and rGO. Moreover, the thermal conductivity, thermal diffusivity, and specific heat capacity were improved gradually depend on the rGO sheets concentration ratio to achieve optimum values of 0.43 W/mK, 0.46 mm^2^/S, and 1.94 MJ/m^3^K at 4%-rGO, respectively. These thermal characteristic variations are prompted by the significant interaction between clay–H_2_O products and oxygen functional groups (epoxides, carboxyl’s, and hydroxyls) of rGO nanosheets. Therefore, these interactions lead to a uniform dispersion of the nanosheets within the clay–rGO composite.

## Data Availability

All data generated or analysed during this study are included in this published article [and its supplementary information files].
